# The evolving spectrum of complex inherited neuropathies

**DOI:** 10.1097/WCO.0000000000001307

**Published:** 2024-07-31

**Authors:** Alexander M. Rossor, Saif Haddad, Mary M. Reilly

**Affiliations:** a Centre for Neuromuscular Diseases, Department of Neuromuscular Diseases, UCL Queen Square institute of Neurology and National Hospital for Neurology and Neurosurgery; b Department of Neurology, Guys and St Thomas’ Hospitals NHS Foundation Trust, UK

**Keywords:** ataxia, peripheral neuropathy, repeat expansion, spasticity

## Abstract

**Purpose of review:**

Inherited peripheral neuropathies can be divided into those diseases in which peripheral neuropathy is the sole or main feature of the disease (Charcot-Marie-Tooth disease) and those in which peripheral neuropathy is just one feature of a more complex syndrome. In recent years there has been a substantial expansion in the number of genes associated with complex neuropathy syndromes.

**Recent findings:**

This review will focus on emerging themes in this group of diseases, namely the increasing number of diseases due to repeat expansions; the emergence of both recessive and dominant negative alleles in the same gene producing a common phenotype and diseases in which there is selective loss of the allele from haematopoietic stem cells making genetic diagnosis on blood derived DNA problematic.

**Summary:**

In this review we provide a practical approach to investigating and diagnosing patients with peripheral neuropathy as part of a complex syndrome and provide an updated table of the genes associated with this group of diseases.

## INTRODUCTION

Healthcare professionals involved in the diagnosis of individuals with inherited peripheral neuropathy have traditionally tested genes associated with diseases in which peripheral neuropathy is the sole or major component of the phenotype. Testing has therefore focused on those genes that have been described in association with Charcot-Marie-Tooth disease (CMT) and the closely related diseases, hereditary sensory neuropathy and distal hereditary motor neuropathy. With the widespread adoption of next generation sequencing into routine clinical practice, it has now become apparent that many complex genetic diseases associated with peripheral neuropathy such as the Hereditary Spastic Paraplegias (HSP) and Spinocerebellar Ataxias can present with peripheral neuropathy. This is relevant as many of the genes responsible for such diseases are absent from inherited neuropathy gene panels. In 2017, we published a clinical approach to these complex neuropathy syndromes [1]. In this review, we revisit our approach and include an up-to-date table of more than 250 genes associated with complex neuropathy syndromes. 

**Box 1:**
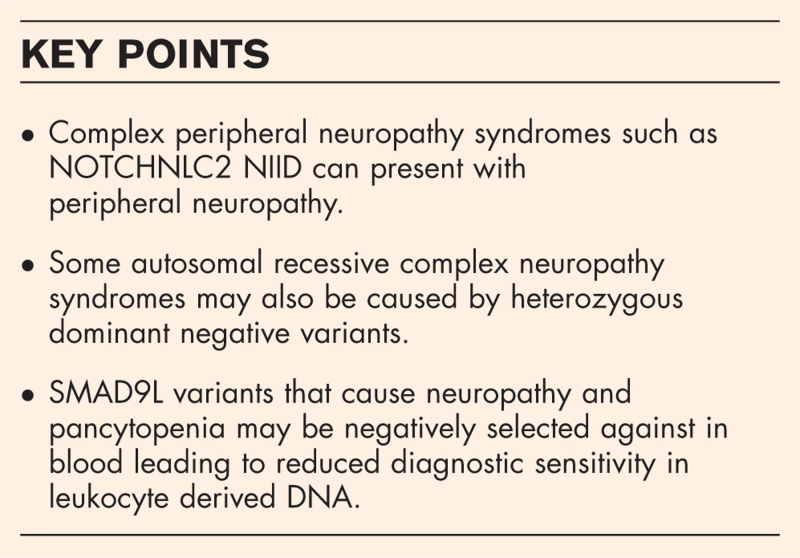
no caption available

## APPROACH TO GENETIC TESTING IN COMPLEX NEUROPATHY SYNDROMES

**FIGURE 1:**
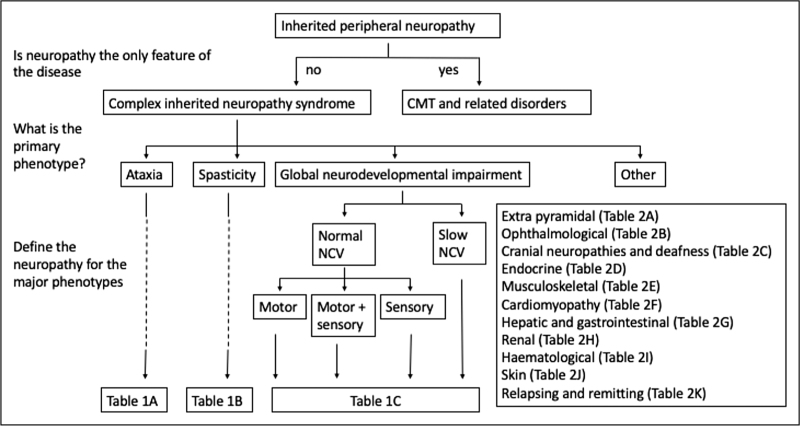
A diagnostic approach for patients with complex inherited neuropathy syndromes.

There are more than 250 genes associated with complex neuropathy phenotypes, many of which are exceptionally rare and reported in only a handful of families. It is therefore unrealistic to expect to have a working knowledge of the phenotypes of all the causative genes. We propose an approach in which the clinician defines the major feature of the nonneuropathy phenotype. In the majority of patients this will be a neurological phenotype of either ataxia, spasticity or global developmental delay (Table 1) although rarer neurological features including optic atrophy, ophthalmoplegia, deafness, myopathy and movement disorders may also be associated with peripheral neuropathy (Table 2). Important nonneurological phenotypes are diverse and include cardiomyopathy, dermatological disease and liver and renal failure (Table 2). Once the predominant phenotype has been defined the neuropathy should then be characterised based on the nerve conduction velocities (NCV) into those with normal or slow conduction. Neuropathies with normal nerve conduction velocities should then be further classified into those which are predominantly sensory, those predominantly motor and those that are mixed (see Fig. 1). Using this approach, one is able to sub classify the many genes associated with complex phenotypes into a workable format (see Tables 1 and 2).

**Table 1:** A summary of the complex inherited neuropathy syndromes with one of the three major core clinical phenotypes of ataxia, spasticity or global neurodevelopmental impairment

Disease (OMIM)	Inheritance	Gene	Clinical description
A. Ataxia and neuropathy syndromes			
Ataxia and sensory predominant axonal neuropathy			
Friedreich ataxia/ FRDA-1 (229300)	AR	*FXN (repeat)*	Early onset ataxia, cardiomyopathy, myelopathy, optic atrophy, sensory axonal neuropathy.
EAOH (208920)	AR	*APTX*	Early onset ataxia, sensory axonal neuropathy, Oculomotor apraxia, Hypoalbuminemia (EAOH)
SCAR1 (606002)	AR	*SETX*	Juvenile onset ataxia, increased α−fetoprotein, nystagmus, cerebellar and pontine atrophy, oculomotor apraxia, sensory axonal neuropathy
Ataxia-telangiectasia (208900)	AR	*ATM*	Childhood onset progressive ataxia, conjunctival telangiectasia, sensory axonal neuropathy, chorea and dystonia, immunodeficiency and increased risk of malignancy, elevated α−fetoprotein
Abetalipoproteinaemia (200100)	AR	*MTTP*	Young onset. Hypocholesterloaemia leading to malabsorption of fat-soluble vitamins (vitamin E), acanthocytes, retinitis pigmentosa, progressive sensory axonal neuropathy
Ataxia with isolated vitamin E deficiency (277460)	AR	*TTPA*	Early onset ataxia and sensory axonal neuropathy similar to Friedreich ataxia, head titubation, normal fat absorption unlike abetalipoproteinaemia, rarely retinitis pigmentosa
Fragile X tremor ataxia syndrome (300623)	AD	*FXTAS* (repeat)	Late onset tremor, ataxia, parkinsonism, sensory axonal neuropathy, middle cerebellar peduncle changes on MRI
SCA27A (609307)	AD	*FGF14*	Learning difficulties, cerebellar ataxia, sensory axonal neuropathy
Galactosialidosis (256540)	AR	*CTSA*	Coarse facies, dwarfism, hearing loss, cherry red macular spot, global developmental delay, ataxia, haemangiomas, vascular abnormalities, rarely sensory axonal neuropathy
CANVAS (614575)	AR	*RFC1 expansion*	Late onset Cerebellar Ataxia, Sensory axonal neuropathy, Vestibular Areflexia Syndrome (CANVAS)
SCA27B (620174)	AD	*FGF14 GAA expansion*	Ataxia, vestibular hypofunction, mild axonal neuropathy
AOA3 (615217)	AR	*PIK3R5*	Oculomotor apraxia, ataxia and sensory axonal neuropathy
Ataxia and sensory-motor axonal neuropathy			
Leukoencephalopathy with brainstem and spinal cord involvement (LBSL) (611105)	AR	*DARS2*	Slowly progressive spasticity, ataxia and dorsal column dysfunction, sensory-motor axonal neuropathy, characteristic MRI findings
Neuropathy, ataxia, retinitis pigmentosa (NARP) (551500)	m8618insTm8993T>G m8993T>Cm9185T>C	*MTATP6*	Ataxia, retinitis pigmentosa, cardiomyopathy, sensory-motor axonal neuropathy
SCAN1 (607250)	AR	*TDP1*	Cerebellar ataxia and sensory-motor axonal neuropathy
Peroxisome biogenesis disorder 6A (214100)	AR	*PEX10*	Failure to thrive, facial dimorphism, agenesis of the corpus callosum, death in first year of life, axonal motor neuropathy, progressive ataxia and sensory-motor axonal neuropathy in adulthood described
Microcephaly, seizures, and developmental delay (MCSZ) (613402)	AR	*PNKP*	Microcephaly, global developmental delay, progressive cerebellar ataxia and atrophy, sensory-motor axonal neuropathy
SCA1 (164400)	AD	*ATXN1 (repeat)*	Adult onset, cerebellar ataxia, spasticity, sensory-motor axonal neuropathy in 40%, occasional choreiform movements
SCA3/MJD (109150)	AD	*ATXN3 (repeat)*	Adult onset, cerebellar ataxia, external ophthalmoplegia, spasticity, extrapyramidal, sensory-motor axonal neuropathy in 50%
SCA5/SCAR14	AD/AR	*SPTBN2*	Cataracts, optic atrophy, cerebellar ataxia, sensory and motor axonal neuropathy PMID:33188499
SCA7 (164500)	AD	*ATXN7 (repeat)*	Adult onset, cerebellar ataxia, pigmentary macular degeneration, sensory-motor axonal neuropathy
SCA10 (603516)	AD	*ATXN10 (repeat)*	Adult onset cerebellar ataxia, sensory-motor axonal neuropathy
SCA12 (604326)	AD	*PPP2R2B (repeat)*	Adult onset cerebellar ataxia, tremor of head and arms, subclinical sensory-motor axonal neuropathy
SCA14 (605361)	AD	*PRKCG*	Usually adult onset isolated cerebellar ataxia. Missense mutation in catalytic domain of exon 11 associated with complex syndrome including cerebellar ataxia, sensory motor axonal neuropathy, parkinsonism, dystonia, myoclonus and pyramidal syndrome.
SCA19 (607346)	AD	*KCDN3*	Prominent ataxic syndrome with possible cognitive decline, movement disorders, and peripheral neuropathy in the late onset forms
SCA23 (610245)	AD	*PDYN*	Cerebellar ataxia, sensory-motor axonal neuropathy
Spinocerebellar ataxia, autosomal recessive 21 (SCAR21) (607982)	AR	*SCYL1*	Early onset ataxia (<1 year) with recurrent episodes of liver failure, sensory-motor axonal neuropathy, cerebellar atrophy
SCAR4 (607317)	AR	*VPS13D*	Saccadic intrusions, acanthocytosis, dyskinesias, psychiatric issues. Neuropathy not characterised.
HLD7 (607694)	AR	*POLR3A*	Adolescent onset progressive spastic ataxia, tremor, involvement of central sensory tracts, dental complications (hypodontia, severe peridontal disease. Bilateral hyperintensities on MRI from the superior cerebellar peduncle to the dentate nucleus / midbrain. ‘Abnormal nerve conduction in 8 out of 14 cases.
SCAN3 (618387)	AR	*COA7*	Cerebellar atrophy, leukoencephalopathy and spinal cord atrophy in some patients. Axonal sensory and motor neuropathy.
AOA4 (616267)	AR	*PNKP*	CMT2, ataxia, microcephaly, seizures, developmental delay
SCAR26 (617633)	AR	*XRCC1*	Ataxia, developmental delay, azoospermia and hypogonadism, myotonia, sensory and motor axonal neuropathy.
SCAR32 (619862)	AR	*PRDX3*	Ataxia and sensory and motor neuropathy
MITCH (618960)	*De novo* dominant	*ACOX1*	Ataxia, sensory motor neuropathy, leukodystrophy, cognitive impairment
Ataxia and motor predominant axonal neuropathy			
SCA2 (183090)	AD	*ATXN2 (repeat)*	Adult onset, slow saccades, ataxia, tremor, parkinsonism, motor>sensory axonal neuropathy in 80%
SCA36 (614153)	AD	*NOP56*	Late adult onset gait ataxia, tongue atrophy and fasciculation, distal motor neuropathy
OHS (304150)	XL	*ATP7A*	Early onset ataxia, spastic tetraparesis, dystonia and axonal motor neuropathy
CMT2Z (616688)	AD	*MORC2*	Congenital onset SMA, cerebellar atrophy and diaphragmatic palsy
CONDCA (618276)	AR	*AGTPBP1*	Early onset cerebellar atrophy, developmental delay, and feeding and respiratory difficulties, severe motor neuronopathy
CONDSIAS (618170)	AR	*ADPRHL2*	Ataxia, spasticity, cognitive impairment / psychosis, motor neuropathy
SCAR8 (610743)	AR	*SYNE1*	Cerebellar atrophy, motor neuropathy in some patients
Ataxia and slow nerve conduction velocity (SNCV)			
Polyneuropathy, hearing loss, ataxia, retinitis pigmentosa and cataracts (PHARC) (612674)	AR	*ABHD12*	Onset 2nd decade, neuropathy with SNCV, sensory neuronal hearing loss, retinitis pigmentosa, spastic paraplegia, ataxia
ARSACS (270550)	AR	*SACS*	Complex neurodegenerative disorder characterized by ataxia, spasticity, neuropathy with SNCV
Ataxia, combined cerebellar and peripheral, with hearing loss and diabetes mellitus ACPHD (616192)	AR	*DNAJC3*	Cerebellar ataxia, neuropathy with SNCV, hearing loss, diabetes mellitus
Cerebrotendinous xanthomatosis (213700)	AR	*CRP27A1*	Adolescent-onset progressive ataxia, myelopathy and dementia, cataracts, low cholesterol, atherosclerosis, xanthomas, soft palate myoclonus, intractable infantile-onset diarrhoea, cerebral white matter lesions on MRI, sensory>motor axonal neuropathy, SNCV described in a minority of patients
Refsum Disease (266500)	AR	*PHYH*	Sensory-motor neuropathy with normal or SNCV, deafness, retinitis pigmentosa, ichthyosis, heart failure, ataxia, raised CSF protein.
DEE32 (616366)	AD	*KCNA2*	Childhood onset spasticity, intellectual disability, ataxia, seizures, sensory and motor SNCV in one family
ATXPC (159550)	AD	*SAMD9L*	Ataxia pancytopenia syndrome, demyelinating neuropathy. Variant may be clonally selected against in blood
CMT1I (619742)	AD	*POLR3B*	De-novo dominant. Ataxia, developmental delay, spasticity and demyelinating neuropathy
B. Spasticity and neuropathy syndromes			
Spasticity and sensory predominant axonal neuropathy			
HSN with spastic paraplegia (256840)	AR	*CCT5*	Severe mutilating sensory neuropathy with spastic paraplegia
SPG61 (615685)	AR	*ARL6IP1*	Childhood onset spastic paraplegia with mutilating, sensory>motor axonal neuropathy
SPG23 (270750)	AR	*DSTYK*	Childhood onset spastic paraplegia, prominent skin pigment abnormalities (vitiligo, hyperpigmentation, diffuse lentigines), premature greying of hair, sensory predominant axonal neuropathy (mild).
SPG35 (612319)	AR	*FA2H*	Childhood onset spasticity, cognitive decline and leukodystrophy. Mild sensory axonal neuropathy on NCS. Epilepsy, dysphagia, dysarthria and dystonia also observed.
SPG79A (620221)/SPG79B (615491)	AD and AR	*UCHL1*	Spasticity, optic atrophy, ataxia, cognitive impairment, sensory axonal neuropathy
SCA25 (608703)	AD	*PNPT1*	Cerebellar ataxia and sensory axonal neuropathy
Spasticity and sensory-motor axonal neuropathy			
SPOAN (609541)	AR	*KLC2*	Early onset spastic paraplegia, congenital optic atrophy, and axonal sensory-motor neuropathy
SPG3A (182600)	AD	*ATL1*	Early onset spastic paraplegia, axonal sensory-motor neuropathy in some patients
SPG7 (607259)	AR	*PGN*	Spastic paraplegia, optic atrophy, ataxia and sensory-motor axonal neuropathy in some patients
SPG10 (604187)	AD	*KIF5A*	Adult onset; spastic paraplegia, axonal sensory-motor neuropathy, rarely parkinsonism and cognitive decline
SPG11 (604360)	AR	*SPG11*	Onset second decade, spastic paraplegia, intellectual disability and cognitive decline, thin corpus callosum, mild cerebellar eye signs, axonal sensory-motor neuropathy, parkinsonism and dystonia, pseudobulbar involvement
SPG15PEX10 (270700)	AR	*ZFYVE26*	As SPG11, but with pigmentary maculopathy
SPG23 (270750)	AR	*DSTYK*	Spastic paraplegia, pigmentary abnormalities, axonal sensory and motor neuropathy
SPG26 (609195)	AR	*B4GALNT1*	Spastic paraplegia, intellectual disability, ataxia, dystonia, axonal sensory-motor neuropathy
SPG28 (09340)	AR	*DDHD1*	Spastic paraplegia, occasionally cerebellar eye signs and subclinical axonal neuropathy
SPG43 (615043)	AR	*C19orf12*	Childhood onset spastic paraplegia and sensory-motor axonal neuropathy, NBIA with optic atrophy, extrapyramidal signs
SPG48 (613647)	AR	*AP5Z1*	Late onset spastic paraplegia and axonal neuropathy (ataxia, dystonia, parkinsonism reported). Thin corpus callosum
SPG54 (615033)	AR	*DDHD2*	Spasticity, developmental delay, ataxia, abnormal eye movements, thin corpus callosum, periventricular white matter lesions, Peripheral neuropathy in some
SPG46 (614409)	AR	*GBA2*	Spastic paraplegia, cognitive decline, thin corpus callosum, ataxia, cataracts, bulbar dysfunction, axonal sensory-motor neuropathy
SPG55 (615035)	AR	*C12ORF6*5	Early onset spastic paraplegia, optic atrophy, intellectual impairment, axonal sensory-motor neuropathy
SPG56 (615030)	AR	*CYP2U1*	Onset 1st decade, spastic paraplegia, rarely dystonia and cognitive impairment, subclinical sensory-motor axonal neuropathy
SPG57 (615658)	AR	*TFG*	Childhood onset spastic paraplegia, sensory-motor axonal neuropathy, optic atrophy
SPAX5 (614487)	AR	*AFG3L2*	Early onset spastic paraplegia, later myoclonic epilepsy, sensory motor axonal neuropathy, ataxia, dystonia
Adult polyglucosan body disease (263570)	AR	*GBE1*	Late onset, cognitive impairment, spasticity, sensory-motor axonal neuropathy, bladder dysfunction, cerebellar and extrapyramidal signs also seen, periventricular white matter abnormalities on MRI
HLD7 (607694)	AR	*POLR3A*	See 1A
SPG76 (616907)	AR	*CAPN1*	Onset 3^rd^ decade, spasticity, dysarthria and ataxia. ‘Peripheral neuropathy’ reported in some individuals
CMT2Z (616688)	AD	*MORC2*	Early onset, pyramidal signs and axonal neuropathy, nonlength dependent weakness, retinitis pigmentosa, developmental delay, deafness, scoliosis, seizures, cataracts, dysmorphisms, nocturnal hypoventilation also reported.
SPAX2 (611302)	AR	*KIF1C*	Onset 1^st^ decade, spasticity, ataxia, cervical dystonia, neuropathy. Variable developmental delay/ occipital/ posterior white matter change.
SPG78 (617225)	AR	*ATP13A2*	Kufor-Rakeb syndrome. Juvenile onset Parkinson's disease, neuronal ceroid lipofuscinosis, cerebellar atrophy, sensory and motor axonal neuropathy.
SPG6 (600363)	AD	*NIPA1*	Spastic paraplegia. Neuropathy infrequently reported
Spasticity and motor predominant axonal neuropathy			
Spinal muscular atrophy, distal (DSMA2) (605726)	AR	*SIGMAR1*	Spastic paraplegia, motor neuronopathy predominantly affecting the extensor muscles of the upper limbs
SPG4 (182601)	AD	*SPAST*	Infantile and adult onset spastic paraplegia, motor axonal neuropathy in some patients
SPG9A (601162) / SPG9B (616586)	AD/AR	*ALDH18A1*	Adolescent and adult onset spastic paraplegia, dysarthria and motor neuronopathy, cataracts, skeletal abnormalities
SPG12 (604805)	AD	*RTN2*	Spastic paraplegia, motor neuropathy seen with homozygous, recessive mutations (MMR, AMR, personal observation)
SPG17 (270685)	AD	*BSCL2*	Silver syndrome, spasticity, motor neuropathy in arms > legs
SPG20/ Troyer syndrome (275900)	AR	*SPG20*	Spasticity, short stature, mental retardation, facial dysmorphism, distal amyotrophy / motor neuropathy
SPG30 (610357)	AR	*KIF1A*	HSP with sensory motor axonal neuropathy +/- cerebellar signs
SPG39 (612020)	AR	*PNPLA6*	Childhood onset of slowly progressive spastic paraplegia; progressive distal motor neuropathy beginning in early through late adolescence
Spasticity and SNCV			
SPG5A (270800)	AR	*CYP7B1*	Childhood to adult onset spastic paraplegia and bladder dysfunction, periventricular white matter abnormalities on MRI, one patient described with SNCV
Adrenoleukodys-trophy (300100)	X-linked	*ABCD1*	Adrenomyeloneuropathy, spastic paraparesis, adrenal insufficiency, axonal sensory-motor neuropathy, sphincter disturbance
Alpha-methylacyl-CoA racemase deficiency (AMACRD) 614307)	AR	*AMACR*	Retinopathy, myelopathy, axonal or SNCV neuropathy, elevated phytanic and pristanic acids
SPG81 (618768)	AR	*SELENOI*	Infantile onset, global developmental delay, spasticity, periventricular white mater signal change on MRI, peripheral neuropathy with SNCV. Seizures and bifid uvula in some affected individuals
CDCBM5 (615763)	AD	*TUBB2A*	Progressive infantile onset spasticity, ataxia and sensory and motor neuropathy. Superior cerebellar vermis atrophy and thin corpus callosum.
C. Global neurodevelopmental impairment and neuropathy syndromes			
Global neurodevelopmental impairment and sensory predominant axonal neuropathy			
Congenital insensitivity to pain	AR	*CLTCL1*	Congenital insensitivity to pain and severe global developmental delay, dysmorphic, delayed myelination on brain MRI
HSAN9 (615031)	AR	*TECPR2*	Global developmental delay, sensory axonal neuropathy, autonomic features, central apnoea / chronic respiratory disease, seizures, encephalopathy
MTDPS7 (271245)	AR	*C10ORF2*	Infantile onset ataxia, PEO, encephalopathy, deafness, seizures and sensory axonal neuropathy
EMPF1 (614388)	AD	*DNM1L*	Developmental delay, global hypotonia and severe ataxia due to axonal sensory neuropathy. Optic atrophy.
Phosphoserine phosphatase deficiency (614023)	AR	*PSPH*	Hereditary sensory neuropathy, osteomyelitis, epilepsy, global developmental delay, responsive to serine.
Global neurodevelopmental impairment and sensory-motor axonal neuropathy			
Giant axonal neuropathy-1 (256850)	AR	*GAN*	Progressive neurodegenerative disorder characterized by spasticity ataxia and sensory-motor axonal neuropathy, kinky/curly hair
Neurodegeneration with brain iron accumulation 2A (NBI2A)/ infantile neuroaxonal dystrophy (INAD) (256600)	AR	*PLA2G6*	Infantile onset, progressive neurodegeneration (tetraplegia, dementia, visual loss) and axonal sensory-motor neuropathy, globus pallidus iron deposition on MRI
CEDNIK syndrome (609528)	AR	*SNAP29*	Cerebral Dysgenesis and severe psychomotor retardation, axonal sensory-motor Neuropathy, Ichthyosis, palmoplantar Keratoderma, fatal by 2^nd^ decade of life.
Pyruvate dehydrogenase E1-alpha deficiency (PDHAD/312170)	X-linked	*PDHA1*	Episodic lactic acidosis, cerebellar ataxia, neurodevelopmental delay and clinical features resembling Leigh syndrome, neuropathy reported (NCV not reported)
Congenital disorder of deglycosylation (615273)	AR	*NGLY1*	Developmental delay, choreoathetosis, alacrimia, seizures, microcephaly, transaminitis, neuropathy
Hypomyelinating leukodystrophy 6 (HLD6/612438)	AD	*TUBB4A*	Early onset, delayed motor development, extrapyramidal movement disorder, spasticity, ataxia, rarely seizures and sensory-motor axonal neuropathy
Mental retardation 9 (601255)	AD	*KIF1A*	Developmental delay, microcephaly, seizures, extrapyramidal disorder, spasticity, cerebellar atrophy, sensory-motor axonal neuropathy
Harel-Yoon syndrome (HAYOS) (617183)	AD	*ATAD3A*	Global developmental delay, optic atrophy, axonal neuropathy, hypertrophic cardiomyopathy
PBD9B (614879)	AR	*PEX7*	Infantile (more severe) variant of Refsum disease, skeletal and facial dysmorphism, global developmental delay
MTDPS5 (612073)	AR	*SUCLA2*	‘Leigh’ like syndrome, deafness, progressive dystonia, mild methylmalonic acidaemia.
PNRIID (618124)	AR	*MCM3AP*	Severe, early onset sensory and motor axonal neuropathy with loss of independent ambulation by the second decade. Mild to moderate intellectual disability
CMT4B3 (615284)	AR	*SBF1*	Infantile onset CMT2, ophthalmoplegia, developmental delay, pyramidal signs, ataxia and cerebellar atrophy.
CMT2Z (616688)	AD	*MORC2*	See 1A and 1B
Phosphoglycerate Kinase 1 Deficiency (300653)	XL	*PGK*	Axonal sensorimotor polyneuropathy, mental retardation, microcephaly, ophthalmoplegia, pes cavus, retinitis pigmentosa, seizures, stroke, parkinsonism, nonspherocytic haemolytic anaemia.
MC4DN11 (619054)	AR	*COX20*	Axonal peripheral neuropathy and static encephalopathy.
DEE80 (618580)	AR	*PIGB*	DOORS syndrome (deafness, onychodystrophy, osteodystrophy, mental retardation, and seizures). Peripheral neuropathy.
DEE44 (617132)	AR	*UBA5*	Severe congenital neuropathy with death in infancy. Epilepsy, movement disorder.
IDDSAPN (619099)	AR	*NEMF*	Speech difficulties, abnormal eye movement, scoliosis intellectual disability and peripheral axonal neuropathy
NEDNMS (619833)	AR	*NRCAM*	Developmental delay, peripheral neuropathy, spasticity. PMID 35108495
CONDMIM (620089)	AR	*LETM1*	Global developmental delay, optic atrophy, sensorineural hearing loss, and cerebellar ataxia, epilepsy, spasticity, myopathy, neuropathy (not characterised) PMID 36055214
PCH10 (615803)	AR	*CLP1*	Failure to develop motor skills, absent/delayed speech, progressive spasticity, epilepsy, sensory and motor axonal neuropathy
WARBM3 (614222)	AR	*RAB18*	Microcephaly, microphthalmia, microcornea, congenital cataracts, optic atrophy, cortical dysplasia, in particular corpus callosum hypoplasia, severe mental retardation, spastic diplegia, and hypogonadism. Sensory and motor axonal neuropathy
PCH2D (613811)	AR	*SEPSECS*	Microcephaly, cortical and cerebellar atrophy, spasticity, seizures, axonal neuropathy.
	De novo	*DHX9*	Cerebellar atrophy, thin corpus callosum, neurodevelopmental delay
Global neurodevelopmental impairment and motor predominant axonal neuropathy			
Hexosaminidase A deficiency (272800)	AR	*HEXA*	Usually infantile onset, developmental delay and cognitive decline, visual loss (“cherry red spot”), motor>sensory neuronopathy, hypometric saccades, adult onset (2nd decade) cases described
Sandhoff disease (268800)	AR	*HEXB*	Indistinguishable from HEXA deficiency
Pontocerebellar hypoplasia type 1B (PCH1B) (614678)	AR	*EXOSC3*	Severe disease often with death in first 5 years, developmental delay, pontocerebellar hypoplasia on MRI, motor neuronopathy
Pontocerebellar hypoplasia (PCH9) (615809)	AR	*AMPD2*	Global developmental delay, spasticity, seizures, dysmorphic facies, axonal neuropathy, agenesis of the corpus callosum and cerebellar hypoplasia on MRI
Spinal muscular atrophy, lower extremity predominant (SMALED1) (158600)	AD	*DYNC1H1*	Congenital onset lower limb motor neuronopathy with contractures, global developmental delay and cerebral dysgenesis in some patients
Spinal muscular atrophy, lower extremity predominant (SMALED2) (615290)	AD	*BICD2*	Congenital onset lower limb motor neuronopathy with contractures, global developmental delay and cerebral dysgenesis in some patients
AAAS (231550)	AR	*AAAS*	Achalasia, addisonianism, alacrima, mental retardation, spastic tetraparesis, bulbospinal motor neuropathy, autonomic neuropathy
Spinal muscular atrophy with progressive myoclonic epilepsy (SMAPME) (159950)	AR	*ASAHI*	Onset first and second decade. Neurodevelopmental delay after onset of seizures. Motor neuronopathy.
NEDHND (617519)	AR	*SPTBN4*	Global developmental delay, congenital myopathy, myopathic facies, axonal motor neuropathy, central deafness.
PEAMO (617207)	AR	*TBCE*	Onset at birth or first year of life. Distal amyotrophy, ataxia, spasticity, optic atrophy, developmental delay.
NMIHBA (617481)	AR	*PRUNE1*	Manitoba Cree population. Microcephaly, brain malformations, infantile contractures, progressive lower and upper motor neuron degeneration.
BIBARS (612292)	AD (maternal imprinting)	*KCNK9*	Birk Barel syndrome. Dysmorphic face. Congenital hypotonia (motor neuropathy). Mild to moderate intellectual disability
D-bifunctional protein (DBP) deficiency (261515)	AR	*HSD17B4*	Peroxisomal disorder. Global developmental delay, seizures, sensorineural hearing loss, cerebellar atrophy, Basal ganglia signal change, leukodystrophy, adrenal insufficiency.
CMT2DD (618036)	AD	*ATP1A1*	Motor axonal neuropathy, spasticity, hypomagnesemia with seizures and intellectual disability
SPG64 (615683)	AR	*ENTPD1*	Global developmental delay, spasticity, motor axonal neuropathy, thin corpus callosum, cerebellar atrophy, signal abnormality posterior limb of the internal capsule
COMNB (619903)	AR	*SLC5A6*	Can present as isolated motor neuropathy, upper limb predominant. Failure to thrive, microcephaly, spasticity, immunodeficiency, osteopenia.
NEDHSCA (616917)	AR	*PIGG*	Mild intellectual disability, cerebellar atrophy, motor neuropathy with conduction block
CONDCA (618276)	AR	*AGTBP1*	Global developmental delay, impaired intellectual development, poor/absent speech, and motor abnormalities, cerebellar atrophy
Martsolf syndrome (212720)	AR	*RAB3GAP2*	Global developmental delay and growth retardation. Spasticity, motor predominant distal neuropathy with SNCV. Microphthalmia, microcornea, cataracts, cryptorchidism, micropenis.
NEDBA (618443)	AD (*de-novo*)/AR	*MAPK8IP3*	Neurodevelopmental delay and motor axonal neuropathy (PMID:37462082/ 30945334)
Global neurodevelopmental impairment and SNCV			
IMNEPD1 (616263)	AR	*PTRH2*	Infantile-onset multisystem disease with intellectual disability, microcephaly, progressive ataxia, sensory neuronal hearing loss, hepatomegaly, pancreatic insufficiency, proximal placement of thumb, SNCV neuropathy.
MEDNIK (609313)	AR	*AP1S1 and AP1B1*	Congenital onset, Mental retardation, Enteropathy (severe congenital diarrhoea), Deafness, sensory-motor Neuropathy with intermediate conduction velocities, Ichthyosis, Keratoderma
Cockayne syndrome (216400/133540)	AR	*ERCC6/ ERCC8*	Dwarfism, optic atrophy, mental retardation, cutaneous photosensitivity, pigmentary retinopathy, deafness, neuropathy with slow conduction velocities
Leigh syndrome variant (256000)	AR	*SURF1*	Leigh syndrome (early onset progressive neurodegeneration of the brain stem, basal ganglia and spinal cord), neuropathy with SNCV
Encephalopathy due to defective mitochondrial and peroxisomal fission 2 (EMPF2) (617086)	AR	*MFF*	Leigh-like syndrome, developmental delay, optic atrophy, seizures, sensory-motor neuropathy with SNCV, Leigh syndrome-like MRI brain (T2 high signal of basal ganglia and sub thalamic nucleus)
Agenesis of the corpus callosum with peripheral neuropathy (ACCPN) (218000)	AR/AD	*SLC12A6*	Mental retardation and progressive neurodegeneration, dysmorphic facies and facial diplegia, agenesis of the corpus callosum, neuropathy with intermediate conduction velocities
Aicardi-Goutieres syndrome	*TREX1 (606609, AD/AR) RNASEH2A (606034), RNASEH2B (AR, 610326), RNASEH2C (AR, 610330), SAMHD1 (AR, 606754), ADAR1 (AR, 146920), IFH1 (AD, 606951)*		Inflammatory syndrome, encephalopathy and psychomotor regression of utero or infantile onset, bilateral striatal necrosis, leukodystrophy, intracranial calcifications, CSF lymphocytosis, spastic paraparesis, rarely neuropathy with SNCV
Leukodystrophy hypomyelination and congenital cataract (HLD5 HCC) (610532)	AR	*FAM126A*	Congenital cataracts, global developmental delay from 1 year, diffuse cerebral hypomyelination on MRI, neuropathy with SNCV
Congenital disorder of glycosylation type 1A (CDG1A) (212065)	AR	*PMM2*	Neonatal onset, leukodystrophy, abnormal serum glycoproteins, mental retardation, hypotonia, ataxia, retinitis pigmentosa, seizures, slowly progressive neuropathy with SNCV, severe infections, hepatic insufficiency and cardiomyopathy.
Metachromatic leukodystrophy (250100)	AR	*ARSA*	Severe late infantile form with mental retardation and severe course. Regression before 30 months; adult onset, psychiatric symptoms, leukodystrophy on MRI, progressive neuropathy with SNCV, optic atrophy
Globoid cell leukodystrophy/ Krabbe (245200)	AR	*GALC*	Spastic paraplegia, developmental delay, optic atrophy; adult onset has spastic paraplegia and sensory-motor axonal neuropathy with slow or normal conduction velocities, MRI shows leukodystrophy
Pelizaeus-Merzbacher disease (PMD) (312080) SPG2 (312920)	X-linked	*PLP1*	Infantile onset, nystagmus, cognitive impairment, spasticity and ataxia, leukodystrophy on MRI, mild multifocal SNCV neuropathy seen with null mutations and more mild phenotype of mild spasticity and ataxia.
HLD2	AR	*GJC2*	Infantile-onset Pelizaeus-Merzbacher disease-like phenotype slowly evolving into a form of complicated hereditary spastic paraplegia with mental retardation, dysarthria, optic atrophy and peripheral neuropathy in adulthood. Leukodystrophy
Cowden syndrome 1 (158350)	AD	*PTEN*	Childhood onset, asymmetric progressive multifocal demyelinating motor neuropathy, macrocephaly, autism spectrum disorder and skin hamartomas
SPG81 (618768)	AR	*SELENOI*	See 1B.
HLD18 (618404)	AR	*DEGS1*	Motor developmental delay, spasticity, cerebellar atrophy and microcephaly, hypomyelination on MRI, scoliosis, neurogenic bladder, enteral nutrition.
NEDMILG (619091)	*AR or de-novo dominant*	*NARS1*	Microcephaly, epilepsy, developmental delay, demyelinating neuropathy
PNSED (616539)	*AR*	*TRMT5*	Global developmental delay, cerebellar abnormalities, severe demyelinating neuropathy
NEDSDV (615075)	*AD*	*CTNNB1*	Spastic paraplegia, exotropia, ataxia and sensory demyelinating neuropathy
NEDSWMA (619026)	*AR*	*HPDL*	Severe, neonatal-onset neurodevelopmental delay with neuroimaging resembling mitochondrial encephalopathy; allelic to SPG83. Slow NCV
IDDPN (619844)	*AR*	*NUDT2*	Infantile onset sensory and motor neuropathy, thin corpus callosum, intellectual impairment. Muscle hypotonia and weakness

AD, Autosomal dominant; AR, Autosomal recessive.

**Table 2:** A summary of the complex inherited neuropathy syndromes with one of the minor 10 clinical phenotypes associated with neuropathy

Disease (OMIM)	Inheritance	Gene	Clinical description
A. Extrapyramidal disease and neuropathy syndromes			
Leukoencephalopathy with dystonia and motor neuropathy (613724)	AR	*SCP2*	Dystonia, hyposmia, azoospermia, motor predominant axonal neuropathy, bilateral thalamic T2 high signal on MRI
MTDPS4B (613662)	AR	*POLG*	SANDO: Sensory Axonal Neuropathy, Dysarthria, Ophthalmoplegia, also parkinsonism and deafness. Also caused by recessive C10orf2 mutations.
Chorea acanthocytosis (200150)	AR	*VPS13A*	Onset 3rd to 5th decade, red cell acanthocytosis and progressive neurodegeneration, seizures, dysarthria, chorea, orofacial dyskinesia, psychiatric disturbance, axonal sensory-motor neuropathy, raised CK
McLeod syndrome (300842)	XL	*XK*	Onset 25–60, acanthocytes and Huntington-like syndrome, also epilepsy, cardiomyopathy, axonal motor neuropathy
CMT2P (614436)	AD/AR	*LRSAM1*	Onset 3^rd^ to 8^th^ decade. Late onset parkinsonism described
DSMA 5 (614881)	AR	*HSJ1*	Onset 2^nd^ decade, motor predominant axonal neuropathy, rarely late onset parkinsonism
Mitochondrial disease	m1095T>C	*MTRNR1 (561000)*	Parkinsonism, deafness, and sensory-motor axonal neuropathy
	AD	*NOTCH2NLC (repeat expansion)*	Essential tremor, cognitive decline, leukodystrophy? axonal sensory and motor neuropathy
SPG10 (604187)	AD	*KIF5A*	See Table 1B
MTDPS5 (612073)	AR	*SUCLA2*	See Table 1C
Siddiqi syndrome (618635)	AR	*FITM2*	Deafness-dystonia syndrome with motor regression and signs of ichthyosis and sensory neuropathy.
Parkinsonism with polyneuropathy (619279)	AD	*UQCRC1*	Levodopa responsive parkinsonism and axonal sensory and motor peripheral neuropathy
B. Ophthalmological and neuropathy syndromes			
Optic atrophy and neuropathy syndromes			
Syndromic optic atrophy (125250)	AD	*OPA1*	Optic neuropathy, PEO, deafness, myelopathy, sensory-motor axonal neuropathy
Costeff syndrome or OPA3-related 3-methylglutaconic aciduria (258501) Optic atrophy and cataracts (165300)	AR/AD	*OPA3*	Infantile optic atrophy, additionally, extra pyramidal disorder (chorea), ataxia, cognitive defects, axonal sensory neuropathy, autonomic neuropathy, pseudo-obstruction
Leber optic atrophy (53500)	Mitochondrial	MT-ND1, ND4, ND6	Optic atrophy, rarely neuropathy, spasticity, ataxia and extrapyramidal signs.
HMSN6B (616505)	AR	*SLC25A46*	Optic atrophy and progressive visual loss in the 1st decade, then spasticity, cerebellar ataxia, sensory-motor axonal neuropathy
BVVLS2 (614707)	AR	*SLC52A2*	Facial and bulbar weakness, sensory ataxia, sensory-motor axonal neuropathy, optic atrophy, sensory neuronal hearing loss
SPOAN (609541)	AR	*KLC2*	See Table 1B
SPG7 (607259)	AR	*PGN*	See Table 1B
SPG43 (615043)	AR	*C19orf12*	See Table 1B
SPG55 (615035)	AR	*C12ORF6*5	See Table 1B
SPG57 (615658)	AR	*TFG*	See Table 1B
Metachromatic leukodystrophy (250100)	AR	*ARSA*	See Table 1C
Krabbe disease (245200)	AR	*GALC*	See Table 1C
EMPF2 (617086)	AR	*MFF*	Leigh-like syndrome, see Table 1C
Cockayne syndrome (216400/133540)	AR	*ERCC6/ERCC8*	See Table 1C
Hexosaminidase A deficiency (272800)	AR	*HEXA*	See Table 1C
Sandhoff disease (268800)	AR	*HEXB*	See Table 1C
HAYOS (617183)	AD	*ATAD3A*	See Table 1C.
HMSN6C (618511)	AR	*PDXK*	Axonal sensory and motor peripheral neuropathy with optic atrophy (31187503). Vitamin B6 responsive.
ANOA (617717)	AR	*FDXR*	Optic and auditory neuropathies. Sensory axonal neuropathy, psychosis, renal tubular acidosis.
MC1DN1 (252010)	AR	*NDUFS6*	Axonal sensory and motor neuropathy, optic atrophy, borderline intellectual disability
Retinitis pigmentosa and neuropathy syndromes			
CMTX5 (311070)	X-linked	*PRPS1*	Hearing loss, retinal dystrophy (optic atrophy), sensory-motor axonal neuropathy
Methylmalonic aciduria and homocystinuria type Cb1c (MMACHC) (277400)	AR	*MMACHC*	Onset infancy to adulthood; thrombotic thrombocytopenia with encephalopathy, myelopathy, renal and pulmonary complications (can be life threatening), retinitis pigmentosa, axonal motor neuropathy; treat with high dose B12
Kearns-Sayre syndrome (530000)	Mitochondrial		Ophthalmoplegia, retinitis pigmentosa, heart block, ptosis
Posterior column ataxia & Retinitis pigmentosa (PCARP / 609033)	AR	*FLVCR1*	Retinitis pigmentosa, sensory ganglionopathy and abnormal posterior columns on MRI
NARP (551500)	Mitochondrial	*MTATP6*	See Table 1A
Refsum Disease (266500)	AR	*PHYH*	See Table 1A
PHARC syndrome (612674)	AR	*ABHD12*	See Table 1A
AMACRD (614307)	AR	*AMACR*	See Table 1B
SPG15 (270700)	AR	*ZFYVE26*	See Table 1B
Cockayne syndrome (216400/133540)	AR	*ERCC6/ERCC8*	See Table 1C
PBD9B (Refsum variant) (614879)	AR	*PEX7*	See Table 1C
Congenital disorder of glycosylation type 1A (212065)	AR	*PMM2*	See Table 1C
Cataracts and neuropathy syndromes			
Congenital cataracts, facial dysmorphism and neuropathy (CCFDN) (604168)	AR	*CTDP1*	Rudari Gypsies, congenital cataracts and microcornea, facial dysmorphism, mild cognitive impairment, neuropathy with SNCV
CMTD1B or CMT2M (606482)	AR	*DNM2*	Intermediate CMT or CMT2, cataracts, ophthalmoplegia, ptosis
Cerebrotendinous xanthomatosis (213700)	AR	*CYP27A1*	See Table 1A
SPG9A (601162) / SPG9B (616586)	AD/AR	*ALDH18A1*	See Table 1B
SPG46 (614409)	AR	*GBA2*	See Table 1B
HLD5 / HCC (610532)	AR	*FAM126A*	See Table 1C
	AR	*PGDH*	Cataracts, developmental delay, ataxia, axonal sensory and motor neuropathy
C. Cranial and peripheral neuropathy syndromes			
FAP-4 (105120)	AD	*GSN*	Corneal lattice dystrophy, cranial neuropathies, cutix laxa
Kearns-Sayre syndrome (530000)	mDNA deletions		Ophthalmoplegia, retinitis pigmentosa, heart block, ptosis
MTDPS8B (612075)	AR	*RRM2B*	PEO, MNGIE, minimal neuropathy
CFEOMA3 (600638)	AD	*TUBB3*	Congenital strabismus, rarely isolated axonal sensory-motor neuropathy, dysgenesis of the corpus callosum, finger and wrist contractures, developmental delay, Kallmann syndrome
SBMA (313200)	XL	*AR*	Motor neuropathy, facial fasciculations, tremor, androgen insensitivity
BVVLS2 (614707)	AR	*SLC52A2*	Facial and bulbar weakness, sensory ataxia, sensory-motor axonal neuropathy, optic atrophy, sensory neuronal hearing loss
BVVLS1 (211530)	AR	*SLC52A3*	Sensory neuronal hearing loss, facial and bulbar weakness, upper limb predominant motor neuropathy
PNMHH (614369)	AR	*MYH14*	Distal myopathy, motor axonal neuropathy, hoarseness, hearing loss
Cowchock syndrome (310490)	X-linked	*AIFM1*	Mental retardation (60%), deafness, slowly progressive sensory and axonal neuropathy from childhood
MELAS (540000)	Mitochondrial	*MTTL1* m3243A>G	Myopathy, deafness, ophthalmoplegia, diabetes, stroke like episodes, predominantly sensory axonal neuropathy
NF2 (101000)	AD	*NF2*	Bilateral acoustic schwannomas. Axonal sensory-motor neuropathy.
Kanzaki disease (609242)	AR	*NAGA*	Adult onset – diffuse angiokeratoma, sensory-neural hearing loss, recurrent episodes of vertigo, sensory-motor axonal neuropathy. Periventricular white matter abnormalities on MRI.
HSN1E (614116)	AD	*DNMT1*	Dementia, deafness and sensory neuropathy
Arthrogryposis distal, with impaired proprioception and touch (617146)	AR	*PIEZO2*	Muscular atrophy with perinatal respiratory distress, ophthalmoplegia, arthrogryposis, and scoliosis
ACPHD (616192)	AR	*DNAJC3*	Deafness. See Table 1A
PHARC syndrome (612674)	AR	*ABHD12*	Deafness. See Table 1A
Refsum Disease (266500)	AR	*PHYH*	Deafness. See Table 1A
PBD9B (Refsum variant) (614879)	AR	*PEX7*	Deafness. See Table 1C
MEDNIK (609313)	AR	*AP1S1*	Deafness. See Table 1C
MTDPS5 (612073)	AR	*SUCLA2*	Deafness. See Table 1C
MTDPS4B (613662)	AR	*POLG*	Deafness. See Table 2A
CMTX5 (311070)	X-linked	*PRPS1*	Deafness. See Table 2B
Perrault Syndrome (233400)	AR	*HSD17B4, TWNK*	Deafness, ovarian dysgenesis, learning difficulties, delayed motor development, cerebellar hypoplasia, peripheral axonal neuropathy
ANOA (617717)	AR	*FDXR*	Deafness. See Table 2B.
D. Endocrinopathy and neuropathy syndromes			
Gonadal dysgenesis with minifascicular neuropathy (607080)	AR	*DHH*	Gonadal dysgenesis, sensory-motor axonal neuropathy
Adrenoleukodystrophy (300100)	XL	*ABCD1*	Adrenal failure, see Table 1B
AAAS (231550)	AR	*AAAS*	Adrenal failure, see Table 1C
Infantile-onset multisystem neurologic, endocrine, and pancreatic disease (IMNEPD) (616263)	AR	*PTRH2*	See Table 1C
SBMA (313200)	X-linked	*AR*	Androgen insensitivity, see Table 2 C
PEPNS (616113)	AR	*DMXL2*	Growth retardation, hypoglycaemia, pyramidal and cerebellar signs. Peripheral neuropathy with slow conduction.
Pituitary hormone deficiency, combined or isolated, 7 (618160)	AR	*RNPC3*	Hypopituitarism and neuropathy
Lipodystrophy, Familial, Partial, Type 9	AR	*PLAAT3*	Lipodystrophy syndrome, demyelinating neuropathy and intellectual disability
E. Musculoskeletal / myopathy and neuropathy syndromes			
Merosin deficient congenital muscular dystrophy (MDC1A) (607855)	AR	*LAMA2*	Congenital muscular dystrophy, mildly slowed PNS conduction, abnormal T2 MRI signal white matter
MFM6 (612954)	AR	*BAG3*	Giant axons on nerve biopsy, myofibrillar myopathy, cardiomyopathy, scoliosis, sensory-motor axonal neuropathy.
Limb girdle muscular dystrophy and neuropathy (181350)	AD	*LMNA*	Limb girdle muscular dystrophy, cardiomyopathy, sensory-motor axonal neuropathy
MERRF (545000)	m8313G>A m8344A>G	*MTTK*	Myoclonic epilepsy, myopathy, lipoma, sensory axonal neuropathy
Multiple acyl-CoA dehydrogenase deficiency (MADD) (231680)	AR	*ETFDH*	Neonatal and late onset forms. hypoglycaemia, metabolic acidosis, and hepatomegaly often preceded by metabolic stress. Muscle involvement in the form of pain, weakness, and lipid storage myopathy also occur. Riboflavin responsive.
HMN2A (158590)	AD	*HSPB8*	Distal hereditary motor neuropathy and proximal myopathy
HMN2B (608634)	AD	*HSPB1*	Distal hereditary motor neuropathy. Myopathic changes on muscle biopsy
Lethal congenital contracture syndrome 1 (253310)	AR	*GLE1*	Micrognathia, pulmonary hypoplasia, loss of anterior horn cells, intrauterine death.
Lethal congenital contracture syndrome 2 (607598)	AR	*ERBB3*	Multiple joint contractures, anterior horn atrophy, death in neonatal period, distended urinary bladder
Lethal congenital contracture syndrome 7 (602346)	AR	*CNTNAP1*	Congenital severe arthrogryposis multiplex congenital, demyelinating neuropathy
Cataracts, Growth Hormone Deficiency, Sensory Neuropathy, Sensorineural hearing loss and skeletal dysplasia (CAGSSS) (616007)	AR	*IARS2*	Spondyloepiphyseal dysplasia, congenital cataracts, nystagmus, dysmorphic facies, sensory neuronal hearing loss, growth hormone deficiency, sensory axonal peripheral neuropathy.
Arthrogryposis distal, with impaired proprioception and touch (617146)	AR	*PIEZO2*	Muscular atrophy with perinatal respiratory distress, ophthalmoplegia, arthrogryposis, and scoliosis
Familial Neuropathic Chronic Itch	AD	*COL6A5*	Idiopathic chronic itch (small fibre neuropathy) and EDS (1 of 3 families)
Arthrogryposis, Distal, Type 5D	AR	*ECEL1*	Contracture of lower limbs, ptosis, scoliosis, hip dislocation, short stature, Thigh MRI, fat replacement sparing rectus femoris and gracilis. EMG in one patient thought to be neurogenic.
PNMHH (614369)	AR	*MYH14*	See Table 2C
NEDHND (617519)	AR	*SPTBN4*	See Table 1C
NEDCPMD (618356)	AR	*NFASC*	Congenital contractures, epilepsy, SNCV neuropathy
SMABF1 (616866)	AR	*TRIP4*	Congenital onset, respiratory failure, slow NCV
LCCS8 (616287)	AR	*ADCY6*	Distal arthrogryposis, hypotonia, respiratory distress, facial diplegia, no motor responses, no myelinated axons
LCCS9 (616503)	AR	*ADGRG6*	Arthrogryposis, prenatal/neonatal death, lack of myelinated axons
LCCS11 (617194)	AR	*GLDN*	Arthrogryposis, neonatal death, widened nodes of Ranvier
AMCNMY (617468)	AR	*LGI4*	Arthrogryposis, foetal death, severe lack of myelinated axons
MFM2 (608810)	AD	*CRYAB*	Myofibrillar myopathy, cataracts
MFM3 (609200)	AD	*MYOT*	myofibrillary myopathy, cardiomyopathy
MFM4 (609452)	AD	*LDB3*	myofibrillary myopathy, cardiomyopathy
MFM5 (609524)	AD	*FLNC*	myofibrillary myopathy, cardiomyopathy
			
F. Cardiomyopathy and neuropathy syndromes			
FAP-1 (105210)	AD	*TTR*	Dysautonomia, cardiac disease carpel tunnel syndrome, painful sensory-motor axonal neuropathy, SNCV may mimic CIDP.
Fabry disease (301500)	X-linked	*GLA*	Angiokeratoma, painful sensory axonal and small fibre neuropathy, cardiomyopathy, renal failure
Mitochondrial complex V deficiency (516070)	m8529G>A	*MTATP8*	Hypertrophic cardiomyopathy, ataxia, PEO, dysarthria, sensory-motor axonal neuropathy
Noonan Syndrome 1 (163950)	AD	PTPN11	Congenital heart defect, multiple lentigines, hypertrophic neuropathy of lumbar plexus
NARP (551500)	mitochondrial	*MTATP6*	See Table 1A
Friedreich ataxia (229300)	AR	*FXN*	See Table 1A
HAYOS (617183)	AD	*ATAD3A*	See Table 1C
McLeod syndrome (300842)	XL	*XK*	See Table 2A
Kearns-Sayre syndrome (530000)	mitochondrial		See Table 2B
MFM6 (612954)	AR	*BAG3*	See Table 2E
COXPD3 (610505)	AR	*TSFM*	Infantile-onset mitochondrial cardiomyopathy, progressing to Leigh syndrome, neuropathy, and optic atrophy. Mild sensory and motor axonal neuropathy
G. Hepatic, gastrointestinal and neuropathy syndromes			
Hepatic			
MTDPS3 (251880)	AR	*DGUOK*	Neonatal liver failure, myopathy, sensory-motor axonal neuropathy
MTDPS6 (256810)	AR	*MPV17*	Corneal opacification, neonatal liver failure, acromutilation, sensory axonal neuropathy, scoliosis, severe motor and sensory axonal neuropathy, cyclical vomiting
SCAR21 (607982)	AR	*SCYL1*	See Table 1A
Tyrosinemia type 1 (276700)	AR	*FAH*	See Table 2H
Gastrointestinal			
MTDPS1 (603041)	AR	*TYMP*	MNGIE: Chronic pseudo-obstruction, Sensory-motor neuropathy with slow conduction (may mimic CIDP), myopathic weakness, cachexia. Leukodystrophy on MRI.
MTDPS4B (613662)	AR	*POLG*	MNGIE: Chronic pseudo-obstruction, axonal sensory ataxic neuropathy, myopathic weakness, cachexia. Normal brain MRI
MTDPS8B (612075)	AR	*RRM2B*	PEO, MNGIE, minimal neuropathy
familial visceral amyloidosis (105200)	AD	*B2M*	Adult onset chronic diarrhoea. Autonomic and sensory-motor axonal neuropathy.
Somatic and autonomic neuropathy	AD	*PRNP*	Autonomic and sensory axonal neuropathy preceding cognitive decline, Chronic diarrhoea.
Goldberg-Shprintzen megacolon syndrome with associated sensory motor axonal neuropathy. (609460)	AR	*KIAA1279*	Intellectual disability, microcephaly, dysmorphic facies, Hirschsprung disease, pachygyria, cerebellar hypoplasia (defect in neural crest migration)
Waardenburg syndrome type 2E (611584) / PWCH (609136)	AD	*SOX10*	Hypopigmentation of the hair and skin, sensory hearing loss, demyelinating neuropathy, dysmyelinating leukodystrophy, developmental delay, spasticity, ataxia, Hirschsprung disease.
AAAS (231550)	AR	*AAAS*	Achalasia. See Table 1C
MEDNIK (609313)	AR	*AP1S1*	Congenital diarrhoea. See Table 1C
Cerebrotendinous xanthomatosis (213700)	AR	*CRP27A1*	Congenital diarrhoea. See Table 1C
FAP-1 (105210)	AD	*TTR*	See Table 2F
Visceral neuropathy, Familial 1, Autosomal Recessive (243180)	AR	*ERBB3*	Hirschsprung, progressive axonal peripheral neuropathy, dysautonomia, Chronic Intestinal Pseudo-obstruction, hypoplasia of olfactory bulbs, external auditory canal agenesis and hearing loss
	AR	*ERBB2*	As above
H. Renal failure and neuropathy syndromes			
FAP-3 (105200)	AD	*APOA1*	Axonal sensory-motor neuropathy similar to TTR FAP, amyloid nephropathy
Action myoclonus-renal failure syndrome (AMRF) (254900)	AR	*SCARB2*	Progressive myoclonic epilepsy with preserved cognition, onset 2nd decade, renal impairment, rarely demyelinating sensory-motor neuropathy (without renal failure)
CMTDIE (614455)	AD	*INF2*	Focal segmental glomerulonephritis and sensory-motor neuropathy with intermediate conduction velocities.
Fabry disease (301500)	X-Linked	*GLA*	See Table 2F
MMACHC (277400)	AR	*MMACHC*	Thrombotic microangiopathy of kidneys (See Table 2I below)
Primary hyperoxaluria type 1 (259900)	AR	*AGXT*	Renal failure and deposition of calcium oxalate crystals in tissues including nerve and muscle. Sensory and motor axonal neuropathy (some slowing)
Lethal congenital contracture syndrome 2 (607598)	AR	*ERBB3*	See 2E. Distended urinary bladder
I. Haematological and immunological neuropathy syndromes			
Methylmalonic aciduria and homocystinuria type Cb1c (MMACHC) (277400)	AR	*MMACHC*	Onset infancy to adulthood; thrombotic thrombocytopenia with encephalopathy, myelopathy, renal and pulmonary complications (can be life threatening), retinitis pigmentosa, axonal motor neuropathy. Treated with high dose vitamin B12.
Chediak-Higashi syndrome (214500)	AR	*LYST*	Partial albinism, immunodeficiency, cerebellar atrophy, sensory-motor axonal neuropathy.
Early-onset chronic axonal neuropathy, strokes, and haemolysis: inherited CD59 deficiency (612300)	AR	*CD59*	Onset 1st and 2nd decade. Haemolytic anaemia, strokes and relapsing immune-mediated demyelinating neuropathy
Autoimmune polyendocrinopathy-candidiasis-ectodermal-dystrophy (APECED) (240300)	AR (rarely AD)	*AIRE*	Multiple autoimmune diseases, classical triad of chronic mucocutaneous candidiasis, hypoparathyroidism and adrenocortical failure. CIDP ‘like illness’ in some patients.
McLeod Syndrome (300842)	X-Linked	*XK*	See Table 2A
J. Skin and connective tissue and neuropathy syndromes			
Xeroderma pigmentosum (278700)	AR	*XPA*	Photosensitivity and increased risk of cutaneous malignancy, global developmental delay, deafness, sensory-motor axonal peripheral neuropathy.
HNARMD (608895)	AD	*FBLN5*	Age related macular degeneration, hyperelastic skin, demyelinating neuropathy also described.
EDS6 (225400)	AR	*PLOD1*	Congenital hypotonia, joint laxity, scleral fragility, susceptibility to large vessel injury, mild sensory-motor axonal neuropathy.
Connective tissue disorder and peripheral neuropathy (130660)	AD	*EMILIN1*	Aortic aneurysm, skin laxity and sensory-motor axonal neuropathy (single family reported)
Refsum disease (266500)	AR	*PHYH*	Ichthyosis. See Table 1A
PBD9B (Refsum variant) (614879)	AR	*PEX7*	Ichthyosis. See Table 1A
Cerebrotendinous xanthomaosis (213700)	AR	*CRP27A1*	Xanthoma. See Table 1A
SPG23 (270750)	AR	*DSTYK*	Skin and hair pigment abnormalities. See Table 1B
CEDNIK syndrome (609528)	AR	*SNAP29*	Icthyosis and palmoplantar keratoderma. See Table 1C.
MEDNIK (609313)	AR	*AP1S1*	Icthyosis and palmoplantar keratoderma. See Table 1C.
Cowden syndrome (158350)	AD	*PTEN*	Skin hamartoma. See Table 1C.
Cockayne syndrome (216400/133540)	AR	*ERCC6/ERCC8*	Cutaneous photosensitivity. See Table 1C
FAP-4 (105120)	AD	*GSN*	Cutis laxa. See Table 2C
Kanzaki disease (609242)	AR	*NAGA*	Angiokeratoma. See Table 2C
Fabry disease (301500)	X-linked	*GLA*	Angiokeratoma. See Table 2C
Phosphoserine Aminotransferase Deficiency (610992)	AR	*PSAT1*	Ichthyosis, arthrogryposis, axonal sensory and motor neuropathy
K. Relapsing complex inherited neuropathy syndromes			
Porphyria, acute intermittent (AIP) (176000)	AD	*HMBS*	Abdominal pain, psychosis, depression, seizures, axonal predominantly motor neuropathy
Coproporphyria (121300)	AD	*CPOX*	Skin photosensitivity and haemolytic anaemia. Can present acutely similar to AIP
Porphyria, variegata (176200)	AD	*PPOX*	Skin photosensitivity. Acute episodes similar to AIP.
Tyrosinemia type 1 (276700)	AR	*FAH*	Infantile or adolescent onset liver disease, renal tubular dysfunction and hypophosphatemic rickets. Acute episodes of neuropathy similar to AIP.
Trifunctional protein deficiency with myopathy and neuropathy (609015)	AR	*HADHA HADHB*	Disorder of mitochondrial beta oxidation of fatty acids. Severe neonatal, infantile and late adolescent onset described, the latter characterised by a progressive myopathy with recurrent rhabdomyloysis and a sensory-motor axonal neuropathy. Abnormal urine organic acids.
Maple syrup urine disease Ib (248600)	AR	*CKDHB*	Metabolic encephalopathy, elevated branched chain amino acids in urine, acute axonal neuropathy
Thiamine metabolism dysfunction syndrome 4 THMD4 (613710)	AR	*SLC25A19*	Acute encephalopathic episodes and paralysis following febrile illness with almost complete recovery. Absent sensory-motor action potential during illness. Bilateral striatal necrosis on MRI. Additional chronic progressive axonal neuropathy
Tangier disease (205400)	AR	*ABC1*	Multifocal relapsing mononeuropathies. Orange tonsils, organomegaly; pain, paresthesia, anaesthesia.
Inherited CD59 deficiency (612300)	AR	*CD59*	See Table 2I
VAIHS (615688)	AR	*ADA2*	Small to medium artery vasculitis
Acute reversible leukoencephalopathy with increased urinary alpha-ketoglutarate (ARLIAK)	AR	*SLC13A3*	Acute encephalopathy, MRI mimicking HIE, raised urinary 2-ketoglutarate. Sensory and motor neuropathy with slow conduction velocity
VEXAS syndrome (301054)	Somatic mutation in haematopoietic progenitor cells	*UBA1*	Polychondritis, myelodysplasia, demyelinating neuropathy.

## RECENT ADVANCES

Since our last review in 2017, there has been a substantial increase in the number of genes associated with complex neuropathy syndromes. This includes an increased number of repeat expansion diseases, the discovery of dominant negative variants for known recessive complex neuropathy syndromes, a new treatable complex syndrome and the discovery of genes such as SMDA9l, which causes an ataxic neuropathy syndrome associated with pancytopenia in which the pathogenic variant is selected against in haematopoietic progenitor cells potentially leading to false negative testing [2].

### Complex neuropathy syndromes due to repeat expansions

#### NOTCH2NLC

Neuronal intranuclear inclusion disease (NIID) is a multisystem neurodegenerative disease that may present with dementia, peripheral neuropathy, autonomic dysfunction, cerebellar ataxia, parkinsonism, seizures or stroke like episodes and fluctuating encephalopathy [3,4]. The disease was described in 1968 and was defined by the presence of eosinophilic intranuclear inclusions on brain biopsy at post mortem that can also be seen in other organs including skin biopsy [5]. In addition to a histopathological signature, brain MRI in NIID commonly reveals leukoencephalopathy on fluid-attenuated inversion recovery (FLAIR) sequences and symmetrical high signal on diffusion weighted imaging (DWI) in corticomedullary junctions [6,7]. Whilst sporadic cases of NIID tend to present with dementia, familial cases cluster into two main phenotypes, a dementia dominant form and a limb weakness dominant form [8].

In 2019, three independent groups identified heterozygous CGG repeat expansions in cohorts of sporadic and familial cases of NIID from Japan and China in the 5’UTR of *NOTCH2NLC*[4,7,8]. These original reports included familial cases presenting with motor and sensory neuropathy and autonomic disturbance. Since the discovery of the genetic cause of NIID, there have been several reports of similar expansions in *NOTCH2NLC* in Chinese and Japanese peripheral neuropathy cohorts [9,10,11^▪▪^]. These include one Taiwanese study in which *NOTCH2NLC* repeat expansions accounted for 10.6% of unassigned CMT2 cases. All patients developed a sensory predominant neuropathy with an average age of onset of 37.1 (range 21–55) [9]. A separate study by Wang *et al.* in an unsolved peripheral neuropathy cohort, found that repeat expansions in *NOTCH2NLC* accounted for 3.52% of Chinese inherited neuropathy patients [12].

The largest study reporting on the prevalence and phenotype of *NOTCH2NLC* related peripheral neuropathy comes from a large case series of inherited peripheral neuropathy in Japan [11^▪▪^]. The cohort comprised 2692 Japanese patients with a diagnosis of CMT, 1783 of whom were without a genetic diagnosis. This cohort was screened for alternative causes with whole exome sequencing and *RFC1* repeat analysis prior to undergoing *NOTCH2NLC* analysis by repeat prime PCR. Using this approach, *NOTCH2NLC* repeat expansions were identified in 26 cases from 22 unrelated families representing 1.2% of genetically undiagnosed Japanese patients with CMT and the seventh most common causative gene in their series excluding CMT1A. The phenotype was characterised by distal wasting and weakness (>90%) with sensory disturbance in 50% of patients. Autonomic dysfunction was common (44%) and characterised by neurogenic bladder, constipation, orthostatic hypotension and erectile dysfunction. Tremor was present in 29% of patients and myoclonus in 4%. Cognitive impairment and ataxia were present in 13 and 9% respectively. Creatine kinase was elevated (mean 559 range 51–1681). Neurophysiology revealed motor nerve conduction velocity slowing in the intermediate range (range 32–49 m/s). Brain MRI was performed in 16 out of 26 patients and was abnormal in 13/16, the most common finding being atrophy and leucoariosis. Only one MRI revealed the characteristic U-fibre high signal on DWI. Both sural nerve and peroneus brevis muscle biopsies revealed positive p62 intranuclear inclusions.

Whilst it is clear that repeat expansions in *NOTCHNLC* are an important cause of inherited peripheral neuropathy in far East populations, similar studies have not been reported in Northern European, North American and African populations. In Caucasian movement disorder patients in the UK, repeat expansions in *NOTCH2NLC* are rare suggesting a possible founder effect in East Asian populations [13].

#### Cerebellar ataxia, neuropathy, and vestibular areflexia syndrome and *RFC1*

Cerebellar ataxia, neuropathy, and vestibular areflexia syndrome (CANVAS) is one of the commonest causes of late onset inherited peripheral neuropathy. It is an autosomal recessive neurodegenerative disease characterised by adult onset sensory neuropathy. Until recently, CANVAS syndrome was thought to be solely caused by biallelic repeat expansions (AAGGG) in the second intron of *RFC1*, which encodes for the large subunit of replication factor C, a 5 subunit DNA polymerase accessory protein [14]. As widespread screening for the repeat expansion has been introduced into clinical practice, a small proportion of individuals with typical CANVAS have been identified that do not carry two (biallelic) repeat expansions.

In two unrelated studies, compound heterozygous variants in *RFC1* comprising one repeat expansion in the second intron of *RFC1* on one allele and a truncating variant in *RFC1* on the other allele were reported in individuals with CANVAS syndrome [15,16^▪▪^]. Although the total number of cases in these two studies was small (*n* < 10), individuals with truncating variants seemed to display a more severe phenotype with two individuals requiring a wheelchair by 50 years of age and one requiring gastrostomy feeding due to severe dysphagia. In one patient the onset of the disease was in the 3^rd^ decade and characterised by spasticity [16^▪▪^].

#### FGF14

Autosomal dominant missense variants in *FGF14* were first described as a cause of SCA27, a rare, early onset ataxic syndrome accompanied by tremor [17]. More recently, a GAA repeat expansion in intron 1 of the same gene, *FGF14* was described as a common cause of autosomal dominant late onset cerebellar ataxia in a cohort of 128 patients [18]. The mean age of onset was in the 6th decade and the phenotype was characterised by gait ataxia, down beat and gaze evoked nystagmus, diplopia, cerebellar dysarthria and cerebellar atrophy on MRI. Peripheral neuropathy was rarely seen in this cohort.

In a study of 45 patients with a combination of cerebellar ataxia and either peripheral neuropathy or bilateral vestibular failure or both (and negative for the RFC1 repeat expansion), screening for the *FGF14* GAA repeat expansion was positive in 43% of patients with ataxia and vestibular failure, 38% with ataxia and neuropathy and 27% with ataxia, neuropathy and vestibular areflexia [19^▪^]. This has led some to claim that heterozygous *FGF14* repeat expansions are a phenocopy of CANVAS syndrome. Whilst this may be technically true, it is important to recognise that in this study the neuropathy was extremely mild and was a mixed motor and sensory neuropathy whereas in CANVAS syndrome due to *RFC1* biallelic repeat expansions, the neuropathy is an early and prominent feature and usually purely sensory. From the available data, it does not appear that repeat expansions in *FGF14* present with peripheral neuropathy unlike CANVAS syndrome.

### Dominant negative variants in recessive complex diseases

Interpreting the pathogenicity of novel variants is dependent on the pattern of inheritance. For example, single heterozygous variants are usually not relevant to a recessive disease. The exception to this is the increasing discovery of heterozygous, often de novo dominant variants in genes such as *SLC12A6, UCHL1* and *NARS1* traditionally associated with autosomal recessive inheritance, which are thought to act through a dominant negative mechanism.

#### SLC12A6

Potassium chloride co-transporters (KCC) are encoded by the SLC12A family of solute carriers, of which *SLC12A6* encodes for the co-transporter protein KCC3 [20]. Recessive variants in *SLC12A6* cause Aldermann's syndrome, a severe early onset motor predominant peripheral neuropathy associated with variable degrees of agenesis of the corpus callosum and developmental delay [21]. In 2016, a child with a severe and progressive peripheral neuropathy was reported in association with a *de novo* heterozygous variant in *SLC12A6*[22]. In the last 4 years, there have been several case series and case reports describing patients with heterozygous variants in *SLC12A6* and a broad phenotype ranging from severe cases with a childhood onset motor predominant axonal neuropathy and developmental delay similar to Alderman's syndrome through to a late onset (5th decade) sensory predominant peripheral neuropathy with no central nervous system involvement [23–26].

#### Ubiquitin C-terminal hydrolase L1

Autosomal recessive missense variants in Ubiquitin C-terminal hydrolase L1 (UCHL1) are a recognised cause of autosomal recessive spastic paraplegia type 79 (SPG79) which is characterised by early onset cerebellar ataxia, spastic paraplegia and optic atrophy [27–29]. In two independent case–control gene burden analyses performed in German and UK cohorts (the latter within the 100 000 genomes project), loss of function heterozygous variants in *UCHL1* were found to be enriched in patients with hereditary ataxia and spastic paraplegia [30]. In the German cohort this was in addition to rare variants in *SPAST, KIF5A* but also *POLR3A*, the latter also having only previously been associated with autosomal recessive disease. The core clinical phenotype included cerebellar ataxia (present in 83%), spasticity in 77% and sensory or sensory motor axonal neuropathy in 52% although only 61% underwent neurophysiology testing. Global developmental delay was a less common feature in dominant UCHL1 disease and the age of onset ranged from childhood to 70 years of age.

#### Asparaginyl-tRNA synthetase

Asparaginyl-tRNA synthetase (NARS1) is a member of the amino acyl tRNA synthetases, a group of enzymes that attach amino acids to their respective tRNA. Recessive loss of function variants have been reported in several AARS genes as a cause of complex hereditary spastic paraplegia including *AARS*, *GARS1* and *DARS2*[31]. This is in contrast to toxic gain of function heterozygous variants that cause a peripheral neuropathy and fall within the CMT spectrum [32]. Recently, both recessive and *de novo* dominant variants in *NARS1* have been described to cause a complex phenotype characterised by severe global (and motor) developmental delay, microcephaly, epilepsy, ataxia and a peripheral neuropathy with slow conduction in 25% [33]. Both recessive and de-novo variants showed a similarly severe phenotype and were associated with loss of amino acetylation activity.

More recently, two variants in two families were reported in association with a pure axonal neuropathy, however a mouse model showed no phenotype in the heterozygous state and it remains uncertain as to whether dominant *NARS1* variants can cause a pure neuropathy [34].

### Treatable complex diseases

*SLC5A6* encodes a nonselective sodium dependent transporter for biotin (B7), pantothenic acid (B5) and lipoic acid. Recessive variants in the gene were originally described in 2017 [35] in individuals presenting in the first few years of life with failure to thrive, developmental delay or regression, seizures, diarrhoea and vomiting, immunodeficiency and osteopenia. As clinical testing has become more widespread, recessive variants have been reported in families with mixed sensory and motor axonal neuropathy with variable conduction velocity slowing associated with optic atrophy and recurrent episodes of pseudo obstruction [36]. In one study, recessive variants in *SLC5A6* were described in three families with onset in the second decade characterised by a motor predominant neuropathy and with subjective improvement or stabilisation with biotin, pantothenic and lipoic acid supplementation [37].

### Ataxia pancytopenia syndrome and loss of pathogenic germ line variants in *SAMD9L* from leukocyte derived DNA

Ataxia- pancytopenia syndrome (ATXPC) due to heterozygous variants in *SAMD9L* was first reported in 2016 [38]. The initial clinical descriptions were of a syndrome of cerebellar ataxia and atrophy with mild pyramidal signs and white matter disease in addition to haematological abnormalities characterised by cytopenia, immunodeficiency and myelodysplasia. More recently a length dependent peripheral neuropathy with slow conduction velocity and pes-cavus has been described and in some families this can be the initial presenting feature [2,39].

Clinical and research genetic testing in the vast majority of cases of peripheral neuropathy is conducted on DNA extracted from white blood cells. This can be problematic for detecting pathogenic *SAMD9L* variants in affected individuals for the reasons I will outline below.

*SAMD9L* is a tumour suppressor gene that inhibits unregulated proliferation of haematological progenitor cells in the bone marrow. Heterozygous pathogenic variants in SAMD9L causing ATXPC exhibit a toxic gain of this function resulting in reduced cell proliferation and cytopenia. This leads to a genetic pressure whereby spontaneous somatic variants including deletions in *SAMD9L* that disrupt or render the inherited pathogenic *SAMD9L* variant null are preferentially selected for that may result in a low level of the mutant allele in blood below the limit of variant calling for many algorithms. This can be overcome by extracting DNA from nonhaematological cells or tissues such as skin. A patient with cytopenia and a peripheral neuropathy, especially with slow NCV in whom gene panel testing on blood has been negative may therefore require further testing on nonhaematological derived DNA.

## CONCLUSION

As more patients are screened for an increasing number of genes, the phenotypes and presentations associated with each gene continue to expand. In the case of inherited peripheral neuropathies, it has become apparent that repeat expansions are emerging as a major cause of complex neuropathy syndromes often in association with cerebellar ataxia. As many of the 250 genes associated with a complex neuropathy syndrome can present with peripheral neuropathy, routine clinical genetic testing in CMT may need to expand to include many of these genes

## Acknowledgements

*We would like to thank Steven Scherer for his assistance in identifying genes associated with complex neuropathy syndromes*

### Financial support and sponsorship

*This research was supported by the National Institute for Health Research University College London Hospitals Biomedical Research Centre. MMR and SH are grateful to the Wellcome Trust (G104817) for funding and MMR also acknowledges funding from the Medical Research Council (MRC MR/S005021/1), National Institutes of Neurological Diseases and Stroke and office of Rare Diseases (U54NS065712 and 1UOINS109403-01), Muscular Dystrophy Association (MDA510281), Charcot Marie Tooth Association (CMTA), Alnylam Pharmaceuticals and Applied Therapeutics.*

### Conflicts of interest

*A.M.R. has received honoraria from Anylam and AstraZeneca pharmaceuticals.*

## REFERENCES AND RECOMMENDED READING

Papers of particular interest, published within the annual period of review, have been highlighted as:

▪ of special interest
▪▪ of outstanding interest

## References

[1] Rossor AM Carr AS Devine H. Peripheral neuropathy in complex inherited diseases: an approach to diagnosis. J Neurol Neurosurg Psychiatry 2017; 88:846–863. 28794150 10.1136/jnnp-2016-313960

[2] Eggermann K Meyer R Begemann M. Clonal elimination of the pathogenic allele as diagnostic pitfall in SAMD9L-associated neuropathy. Genes (Basel) 2022; 13: [Online ahead of print]. 10.3390/genes13122356 PMC9778166 36553623

[3] Zhang T Bao L Chen H. Review of phenotypic heterogeneity of neuronal intranuclear inclusion disease and NOTCH2NLC -related GGC repeat expansion disorders. Neurol Genet 2024; 10: [Online ahead of print]. 10.1212/NXG.0000000000200132 PMC10997217 38586597

[4] Tian Y Wang JL Huang W. Expansion of human-specific GGC repeat in neuronal intranuclear inclusion disease-related disorders. Am J Hum Genet 2019; 105:166–176. 31178126 10.1016/j.ajhg.2019.05.013 PMC6612530

[5] Sone J Tanaka F Koike H. Skin biopsy is useful for the antemortem diagnosis of neuronal intranuclear inclusion disease. Neurology 2011; 76:1372–1376. 21411744 10.1212/WNL.0b013e3182166e13

[6] Sone J Mori K Inagaki T. Clinicopathological features of adult-onset neuronal intranuclear inclusion disease. Brain 2016; 139:3170–3186. 27797808 10.1093/brain/aww249 PMC5382941

[7] Ishiura H Shibata S Yoshimura J. Noncoding CGG repeat expansions in neuronal intranuclear inclusion disease, oculopharyngodistal myopathy and an overlapping disease. Nat Genet 2019; 51:1222–1232. 31332380 10.1038/s41588-019-0458-z

[8] Sone J Mitsuhashi S Fujita A. Long-read sequencing identifies GGC repeat expansions in NOTCH2NLC associated with neuronal intranuclear inclusion disease. Nat Genet 2019; 51:1215–1221. 31332381 10.1038/s41588-019-0459-y

[9] Liao YC Chang FP Huang HW. GGC repeat expansion of NOTCH2NLC in Taiwanese patients with inherited neuropathies. Neurology 2022; 98:E199–206. 34675106 10.1212/WNL.0000000000013008

[10] Yu J Luan X hua Yu M. GGC repeat expansions in NOTCH2NLC causing a phenotype of distal motor neuropathy and myopathy. Ann Clin Transl Neurol 2021; 8:1330–1342. 33943039 10.1002/acn3.51371 PMC8164861

[11▪▪] Ando M Higuchi Y Yuan JH. Clinical phenotypic diversity of NOTCH2NLC -related disease in the largest case series of inherited peripheral neuropathy in Japan. J Neurol Neurosurg Psychiatry 2023; 94:622–630. 36948577 10.1136/jnnp-2022-330769

[12] Wang H Yu J Yu M. GGC repeat expansion in the NOTCH2NLC gene is associated with a phenotype of predominant motor–sensory and autonomic neuropathy. Front Genet 2021; 12: [Online ahead of print]. 10.3389/fgene.2021.694790 PMC8293674 34306035

[13] Yau WY Vandrovcova J Sullivan R. Low prevalence of NOTCH2NLC GGC repeat expansion in white patients with movement disorders. Mov Disord 2021; 36:251–255. 33026126 10.1002/mds.28302 PMC8436747

[14] Cortese A Simone R Sullivan R. Biallelic expansion of an intronic repeat in RFC1 is a common cause of late-onset ataxia. Nat Genet 2019; 51:649–658. 30926972 10.1038/s41588-019-0372-4 PMC6709527

[15] Benkirane M Da Cunha D Marelli C. RFC1 nonsense and frameshift variants cause CANVAS: Clues for an unsolved pathophysiology. Brain 2022; 145:3770–3775. 35883251 10.1093/brain/awac280

[16▪▪] Ronco R Perini C Currò R. Truncating variants in RFC1 in cerebellar ataxia, neuropathy, and vestibular areflexia syndrome. Neurology 2023; 100:E543–E554. 36289003 10.1212/WNL.0000000000201486 PMC9931080

[17] Van Swieten JC Brusse E De Graaf BM. A mutation in the fibroblast growth factor 14 gene is associated with autosomal dominant cerebral ataxia. Am J Hum Genet 2003; 72:191–199. 12489043 10.1086/345488 PMC378625

[18] Pellerin D Danzi MC Wilke C. Deep intronic FGF14 GAA repeat expansion in late-onset cerebellar ataxia. N Engl J Med 2023; 388:128–141. 36516086 10.1056/NEJMoa2207406 PMC10042577

[19▪] Pellerin D Wilke C Traschütz A. Intronic FGF14 GAA repeat expansions are a common cause of ataxia syndromes with neuropathy and bilateral vestibulopathy. J Neurol Neurosurg Psychiatry 2023; 95:175–179. 10.1136/jnnp-2023-331490 PMC10850669 37399286

[20] Kahle KT Khanna AR Alper SL. K-Cl cotransporters, cell volume homeostasis, and neurological disease. Trends Mol Med 2015; 21:513–523. 26142773 10.1016/j.molmed.2015.05.008 PMC4834970

[21] Howard HC Mount DB Rochefort D. The K-Cl cotransporter KCC3 is mutant in a severe peripheral neuropathy associated with agenesis of the corpus callosum. Nat Genet 2002; 32:384–392. 12368912 10.1038/ng1002

[22] Kahle KT Flores B Bharucha-Goebel D. Peripheral motor neuropathy is associated with defective kinase regulation of the KCC3 cotransporter. Sci Signal 2016; 9:ra77. 27485015 10.1126/scisignal.aae0546 PMC5506493

[23] Bogdanova-Mihaylova P McNamara P Burton-Jones S Murphy SM. Expanding the phenotype of SLC12A6-associated sensorimotor neuropathy. BMJ Case Rep 2021; 14:12–15. 10.1136/bcr-2021-244641 PMC8552160 34706912

[24] Løseth S Høyer H Le KM. Late-onset sensory-motor axonal neuropathy, a novel SLC12A6-related phenotype. Brain 2023; 146:912–922. 36542484 10.1093/brain/awac488 PMC9976957

[25] Ando M Higuchi Y Yuan J. Novel heterozygous variants of SLC12A6 in Japanese families with Charcot–Marie–Tooth disease. Ann Clin Transl Neurol 2022; 9:902–911. 35733399 10.1002/acn3.51603 PMC9268887

[26] Shi J Zhao F Pang X. Whole-exome sequencing identifies a heterozygous mutation in SLC12A6 associated with hereditary sensory and motor neuropathy. Neuromuscul Disord 2021; 31:149–157. 33323309 10.1016/j.nmd.2020.11.002

[27] Bilguvar K Tyagi NK Ozkara C. Recessive loss of function of the neuronal ubiquitin hydrolase UCHL1 leads to early-onset progressive neurodegeneration. Proc Natl Acad Sci USA 2013; 110:3489–3494. 23359680 10.1073/pnas.1222732110 PMC3587195

[28] Das Bhowmik A Patil SJ Deshpande DV. Novel splice-site variant of UCHL1 in an Indian family with autosomal recessive spastic paraplegia-79. J Hum Genet 2018; 63:927–933. 29735986 10.1038/s10038-018-0463-6

[29] Rydning SL Backe PH Sousa MML. Novel UCHL1 mutations reveal new insights into ubiquitin processing. Hum Mol Genet 2016; 26:1031–1040. 10.1093/hmg/ddw391 28007905

[30] Park J Tucci A Cipriani V. Heterozygous UCHL1 loss-of-function variants cause a neurodegenerative disorder with spasticity, ataxia, neuropathy, and optic atrophy. Genet Med 2022; 24:2079–2090. 35986737 10.1016/j.gim.2022.07.006

[31] Lynch DS Rodrigues Brandão De Paiva A Zhang WJ. Clinical and genetic characterization of leukoencephalopathies in adults. Brain 2017; 140:1204–1211. 28334938 10.1093/brain/awx045 PMC5405235

[32] Wei N Zhang Q Yang X-L. Neurodegenerative Charcot–Marie–Tooth disease as a case study to decipher novel functions of aminoacyl-tRNA synthetases. J Biol Chem 2019; 294:5321–5339. 30643024 10.1074/jbc.REV118.002955 PMC6462521

[33] Manole A Efthymiou S O’Connor E. De novo and bi-allelic pathogenic variants in NARS1 cause neurodevelopmental delay due to toxic gain-of-function and partial loss-of-function effects. Am J Hum Genet 2020; 107:311–324. 32738225 10.1016/j.ajhg.2020.06.016 PMC7413890

[34] Beijer D Marte S Li JC. Dominant NARS1 mutations causing axonal Charcot–Marie–Tooth disease expand NARS1-associated diseases. Brain Commun 2024; 6:1–9. 10.1093/braincomms/fcae070 PMC10943570 38495304

[35] Subramanian VS Constantinescu AR Benke PJ Said HM. Mutations in SLC5A6 associated with brain, immune, bone, and intestinal dysfunction in a young child. Hum Genet 2017; 136:253–261. 27904971 10.1007/s00439-016-1751-x PMC5263180

[36] Montomoli M Vetro A Tubili F. A novel SLC5A6 homozygous variant in a family with multivitamin-dependent neurometabolic disorder: phenotype expansion and long-term follow-up. Eur J Med Genet 2023; 66:104808. 37391029 10.1016/j.ejmg.2023.104808

[37] Mansour-Hendili L Gitiaux C Harion M. Recurrent “outsider” intronic variation in the SLC5A6 gene causes severe mixed axonal and demyelinating neuropathy, cyclic vomiting and optic atrophy in 3 families from Maghreb. Front Genet 2024; 15:1–7. 10.3389/fgene.2024.1352006 PMC10859498 38348452

[38] Chen D-H Below JE Shimamura A. Ataxia-pancytopenia syndrome is caused by missense mutations in SAMD9L. Am J Hum Genet 2016; 98:1146–1158. 27259050 10.1016/j.ajhg.2016.04.009 PMC4908176

[39] Paucar M Tesi B Eshtad S. Adult-onset ataxia with neuropathy and white matter abnormalities due to a novel SAMD9L variant. Neurol Genet 2021; 7:4–6. 10.1212/NXG.0000000000000628 PMC8554712 34722875

